# Substitution of Ag for Cu in Cu_2_ZnSn(S,Se)_4_: Toward Wide Band Gap Absorbers with Low Antisite Defects for Thin Film Solar Cells

**DOI:** 10.3390/nano10010096

**Published:** 2020-01-03

**Authors:** Yanjie Wu, Yingrui Sui, Wenjie He, Fancong Zeng, Zhanwu Wang, Fengyou Wang, Bin Yao, Lili Yang

**Affiliations:** 1 Key Laboratory of Functional Materials Physics and Chemistry of the Ministry of Education, Jilin Normal University, Siping 136000, China; yanjiewu1993@163.com (Y.W.); jlnuhwj@163.com (W.H.); ZENG740183899@163.com (F.Z.); wangzhanwu@126.com (Z.W.); wfy@jlnu.edu.cn (F.W.); yanglili1998@126.com (L.Y.); 2 State Key Laboratory of Superhard Materials and College of Physics, Jilin University, Changchun 130012, China

**Keywords:** (Cu_1-x_Ag_x_)_2_ZnSn(S,Se)_4_, thin films, photoelectric performance, antisite defects, sol–gel, solar cells

## Abstract

Cation substitution is a promising approach to reduce the antisite defects and further improve the efficiency of Cu_2_ZnSn(S,Se)_4_ (CZTSSe) cells. In this paper, silver (Ag) has been introduced into Cu_2_ZnSn(S,Se)_4_ (CZTSSe) thin film to replace Cu partially and form (Cu_1-x_Ag_x_)_2_ZnSn(S,Se)_4_ (0 ≤ x ≤ 1) (CAZTSSe) alloy films by combination of solution method and a rapid annealing technique. The fundamental properties of the mixed Ag-Cu kesterite compound are systematically reported as a function of the Ag/(Ag+Cu) ratio. The results show that band gap of kesterite CAZTSSe is incessantly increased by adjusting the Ag doping content, indicating that the CAZTSSe alloy film is a potentially applicable bandgap grading absorption layers material to obtain higher CZTSSe device. Furthermore, CAZTSSe alloy films with better electrical performance were also obtained by adjusting the Ag content during film fabrication. Finally, we also observed an increment in open circuit voltage (Voc) by 160 mV and an accompanying rise in device efficiency from 4.24 to 5.95%. The improvement is correlated to the improved grain size and decreased antisite defects of Cu instead of Zn site (Cu_Zn_) in the lattice. The Voc enhancement evidences that the solution method is facile and viable to achieve proper cation substitution toward higher efficiency kesterite solar cells. In addition, the CAZTSSe cell also displays better charge collection performance because of the higher fill factor (FF) and power conversion efficiency (PCE). Therefore, it can be concluded that the doping of Ag is a potentially appropriate method to reduce the Cu_zn_ antisite defects of CZTSSe and improve efficiency of CZTSSe device.

## 1. Introduction

Cu_2_ZnSn(S,Se)_4_ (CZTSSe) film has been regarded as a promising film because of the earth abundant elements and favorable optoelectronic properties, which may substitute Cu(In,Ga)Se_2_ (CIGS) thin film and other inorganic films [1,2,3,4,5,6,7]. Up to now, the best reported power conversion efficiency (PCE) of 12.6% for CZTSSe solar cells distinctly lags theoretically predictive values and the PCE of CIGS solar cells [8]. It is well-known that the PCE of CZTSSe devices is primarily limited by the lower open-circuit voltage (V_oc_), reasons of which have been the subject of debate fiercely [9,10]. A major interpretation is CZTSSe thin films encountering in serious band tailing because of a high concentration of defects which can be responsible for an imperative component of the V_oc_ loss [9]. It has been also reported that the band tailing of CZTSSe film is dramatically more severe than that of CIGS film [11]. Thus, solving this problem is considered as a hopeful method to improve the PCE of CZTSSe solar cells. Some literatures have indicated that the occupation of Cu atoms in the Zn lattice sites producing the antisite defects of the occupation of Cu atoms in the Zn lattice sites (Cu_Zn_) should be avoided because they can generate band tailing, deep surface or bulk defects, which limited the V_oc_ of devices [12,13]. Because of the similar ionic radius of Cu and Zn, the formation energy of the Cu_Zn_ antisite defects is relatively low. Therefore, the CZTSSe film exists a high concentration of Cu_Zn_ antisite defects [12]. The replacement of cation with much bigger size than Cu can improve the formation energy of Cu_Zn_ antisite defects [14]. In addition to inhibiting the formation energy of Cu_Zn_ antisite defects, the replacement of cations can also adjust band gap of the CZTSSe film and make it better match to CdS layer [15,16]. Therefore, developing an element to replace Cu or Zn to decrease the band tailing in CZTSSe absorber thin film is of great significance.

Disparate elements have been attempted to substitute cations in CZTSSe thin films. Lately, Giraldo et al. improved the PCE of CZTSe from 7.0 to 10.1% by replacing Sn by Ge ion [17]. It has also been reported that PCE of CZTS device could be improved dramatically from 5.3% to 9.24% by the substitution Zn by Cd cations, implying the cation substitution exists in prodigious capacity for enhancing PCE of CZTSSe device [15], whereas no distinct improvement in V_oc_ for CZTSSe solar cells was observed. The theoretical results indicated that the Ag replacement of Cu in CZTSSe is a valid way to reduce the antisite defect concentration and increase the band gap for CZTSSe absorber layer to an optimized value [13]. Several experiments have reported that the Ag doping of CZTSSe on the device properties has positive effects [18,19]. More recently, Gershon et al. obtained the mixed alloy CAZTSe by co-evaporation [20]. A device efficiency of 10.2% and a 37 mV enhancement in Voc has been achieved. Guchhait et al. obtained the mixed alloy CAZTSe by sol–gel method [21]. The significant increment in Voc by 50 mV and an accompanying rise in device efficiency from 4.9% to 7.2% were obtained [21]. However, there are a few reports on performance of CAZTSSe film based on direct solution coating of Ag containing precursors. Sol–gel technique is a preferable method, which can accurately control the content of cations and doped elements in the precursor solution and can easily obtain uniformly distributed CAZTSSe film. In this paper, it has been demonstrated that Ag replacement of Cu can be expediently implemented in CAZTSSe thin films across the full Ag composition range by sol–gel technique. Structure, optical, and electrical properties of CAZTSSe films was systematically researched. As is known to all, CZTSSe solar cells with highly PCE need a high annealing temperature that induces Sn element loss by gaseous SnSe_2_, which causes the increase of impurity phase and defects [22]. In this work, the high quality CAZTSSe films were synthesized at a relatively low annealing temperature for the first time. Meanwhile, the influence of doped Ag on the CAZTSSe device performance was investigated in detail. Finally, CAZTSSe device of 5.95% PCE had been obtained with 10% Ag incorporation, which is achieved by about 160 mV improvements in Voc. 

## 2. Experimental 

The solution was prepared by dissolving Cu(CH_3_COO)_2_, AgNO_3_, Zn(CH_3_COO)_2_, SnCl_2_, and CH_4_N_2_S and 2-methoxyethanol. The molar ratio of (Cu+Ag), Zn, Sn, and S in the solution is 2.125:1.5:1:8 respectively. And the Ag/(Cu+Ag) ratios were changed from 0 to 1. The solution was spin coated on substrates for CAZTS thin films. The spin coating process was made at 3000 r/min for 30 s and followed with drying on the hot plate at 300 °C. The spin coating and sintering processes were repeated to get CAZTS film. To obtain the CAZTSSe thin films, CAZTS films together with 0.2 g selenium powder were loaded into a graphite box and then annealed in a rapid thermal processing furnace at 480 °C for 10 min at Ar atmosphere.

The crystal structures of CAZTSSe samples were measured by X-ray diffractometer (XRD, Rigaku Corporation, Tokyo, Japan) with Cu *Kα* (λ = 0.15406 nm) source. Raman spectroscope was characterized by a Renishaw system excited with 514 nm wavelength. The composition and valence state of elements for CAZTSSe films were measured by X-ray photoelectron spectroscopy (XPS) (XPS, Thermo Fisher Scientific, Waltham, MA, USA) with an Al *Kα* radiation source (ESCALAB MARK II, VG Inc.). The scanning electron microscope (SEM) image was measured by a Hitachi S-4800 equipped with an energy-dispersive X-ray spectroscopy (EDS) system (Hitachi S-4800, JEOL Ltd., Tokyo, Japan). The electrical performance was characterized by a Hall-effect measurement system at room temperature. The optical performance was carried out by using UV-Vis-near-infrared (NIR) (UV-3101PC, Tokyo, Japan). The photocurrent density dependence on the voltage (J–V) were measured under AM 1.5 G simulated sunlight illumination (Model 91160, Newport, Irvine, American). The spectral response was taken by an EQE measurement system (QEX10, Newport, Irvine, American).

## 3. Results and Discussion

**Figure 1:**
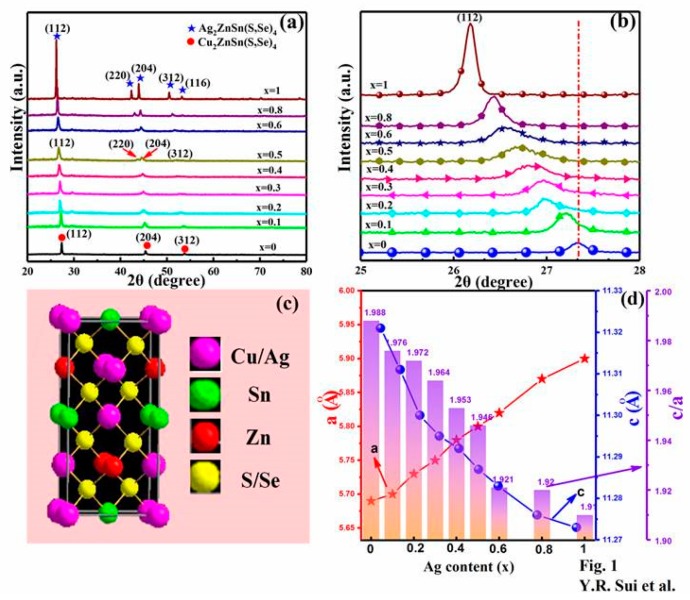
(**a**) X-ray diffractometer (XRD) spectra of CAZTSSe (0 ≤ x ≤ 1) alloy thin films. (**b**) Enlarged view of the corresponding (112) diffraction peaks of the CAZTSSe (0 ≤ x ≤ 1) alloy thin films. (**c**) The structure cell of the kesterite CAZTSSe. (**d**) Lattice parameters a and c for the CAZTSSe (0 ≤ x ≤ 1) alloy thin films.

Crystal structure and phase composition of the CAZTSSe films with different Ag contents were performed by XRD. Figure 1a shows the XRD patterns of CAZTSSe alloy thin films as the x changed from 0 to 1. The XRD peaks displayed in Figure 1a consist of diffraction peaks of CZTSSe and Ag_2_ZnSn(S,Se)_4_ (AZTSSe), which are marked with circulars and stars, respectively. It can be seen from Figure 1a that the CAZTSSe (0 ≤ x ≤ 0.4) films exhibited dominant peaks from (112), (204), and (312) planes, which could be in accordance with tetragonal kesterite-type structure of CZTSSe [23,24]. In addition to CZTSSe diffraction peaks, peaks related to either metallic Ag, Cu, or their complex oxide were not observed for CAZTSSe (0 ≤ x ≤ 0.4) films, indicating doped-Ag has not altered the basic structure of CZTSSe and is not involved in the formation of other impurity atoms, that is, the Ag^+^ had been incorporated into CZTSSe lattice. When x increased to 0.5, the CAZTSSe were mixture of two crystalline phases including CZTSSe and AZTSSe. And for the CAZTSSe (0.6 ≤ x ≤ 0.8), the diffraction peak of (204) for CZTSSe film split into (220) and (204) diffractions of AZTSSe, and the diffraction peak of (312) for CZTSSe film into (312) and (116) diffractions of AZTSSe clearly were observed because of the increase in lattice parameters. At the same time, the diffraction peak intensity of CZTSSe decreased and that of AZTSSe increased as the x increased, indicating that the relative content of the CZTSSe decreased, whereas that of the AZTSSe increased. Similar phenomena have been reported in previous literature [25]. As the x increased to 1, some diffraction peaks were observed, which were ascribed to diffraction of (112), (220), (204), (312), and (116) planes of AZTSSe with tetragonal kesterite-type structure [25].

Figure 1b displays the amplified views of (112) diffraction peak of CAZTSSe (0 ≤ x ≤ 1) thin films in a diffraction angle 2θ between 25° and 28°. It was obvious that the (112) diffraction peak gradually moved toward lower angles as the Ag content increased, indicating that the lattice constant enlarged. The lattice constants of the CAZTSSe films were closely related to the ionic size. The ionic size of the Ag^+^ is 1.14 Å but it is 0.77 Å for Cu^+^ in CZTS film [25]. Therefore, it could be deduced that Ag mainly occupied Cu sites for CAZTSSe film. According to the results of XRD, the structure cell of the kesterite CAZTSSe is listed in Figure 1c. It could be found that Cu^+^ ions and Ag^+^ ions took up the same location in the structure cell. The lattice parameters a and c for CAZTSSe alloy thin films were calculated and presented in Figure 1d. It was found that the a-axis lattice parameter linearly increased, the c-axis lattice parameter decreased, the c/a ratio decreased with increasing Ag content. It has been reported that that the value of c/a for CZTSSe compounds were higher than that of AZTSSe [25]. According to the result of Figure 1d, we found that the c/a values for CZTSSe was higher than that of all CAZTSSe, and the c/a values gradually decreased with the increase of Ag content. Therefore, it was proved that Ag^+^ ions had been successfully doped into CZTSSe lattice and formed homogeneous CAZTSSe film again. 

**Figure 2:**
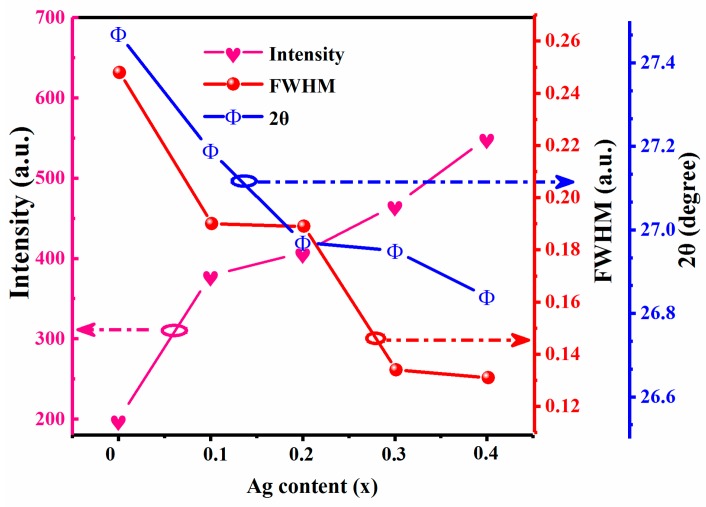
Variation in diffraction angle 2θ, the full-width at half-maximum (FWHM) and intensity of (112) peaks against various Ag contents for CAZTSSe (0 ≤ x ≤ 0.4) alloy thin films.

To describe the effect of Ag on structure performances of CAZTSSe with kesterite structure, the peak intensity, the full-width at half-maximum (FWHM), and diffraction angle 2θ of (112) peak in XRD were obtained and are summarized in Figure 2. Figure 2 shows the peak intensity, FWHM, and 2θ of (112) peak in CAZTSSe (0 ≤ x ≤ 0.4) films, respectively. The intensity of (112) peak was obviously enhanced, indicating that the crystalline quality of CAZTSSe thin films was improved as the Ag content was increased, as shown in Figure 2. Correspondingly, FWHM of (112) peak has been decreased significantly. Therefore, it can be further demonstrated that the CAZTSSe films prepared at higher Ag doping content process better crystalline quality. It is also observed that the gradual decreasing tendency in 2θ angle of (112) with increasing Ag content was revealed. It is possibly because of the fact that the substitution of smaller Cu^+^ by larger Ag^+^ took place in CAZTSSe film. 

**Figure 3:**
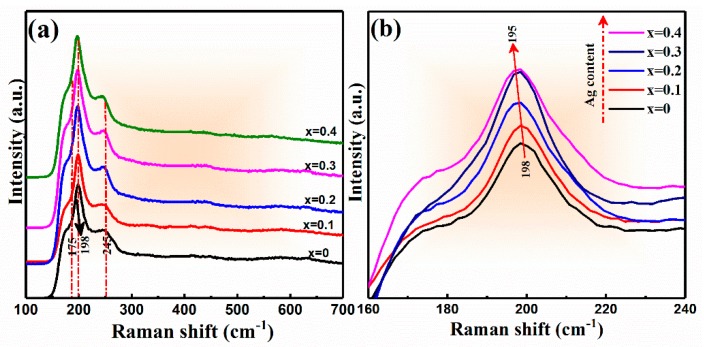
(**a**) Raman spectra of the CAZTSSe (0 ≤ x ≤ 0.4) alloy thin films. (**b**) Enlarged view of the main Raman peaks of A1 mode for CAZTSSe (0 ≤ x ≤ 0.4) alloy thin films.

To identify the effect of Ag on Raman spectra of CAZTSSe (0 ≤ x ≤ 0.4), Raman spectroscopy measurement was recorded for CAZTSSe (0 ≤ x ≤ 0.4) films, as displayed in Figure 3a. For Raman spectra of CAZTSSe films, three Raman peaks situated at 175, 198, and 245 cm^–1^ were obviously found, they were attributed to the A1 vibrational mode from kesterite structure. This is consistent with the reported literatures [26]. The change of the main Raman peak position for CAZTSSe (0 ≤ x ≤ 0.4) films is displayed in Figure 3b. Compared with pure CZTTSe film, the CAZTSSe films had a slight peak shifted toward the lower wavenumber. This could be accounted for the doping of Ag into the CZTSSe lattice and led to change of Raman peak position, indicating the influence of the replacement of Cu^+^ by the Ag^+^, which was consistent with the results of XRD.

**Figure 4:**
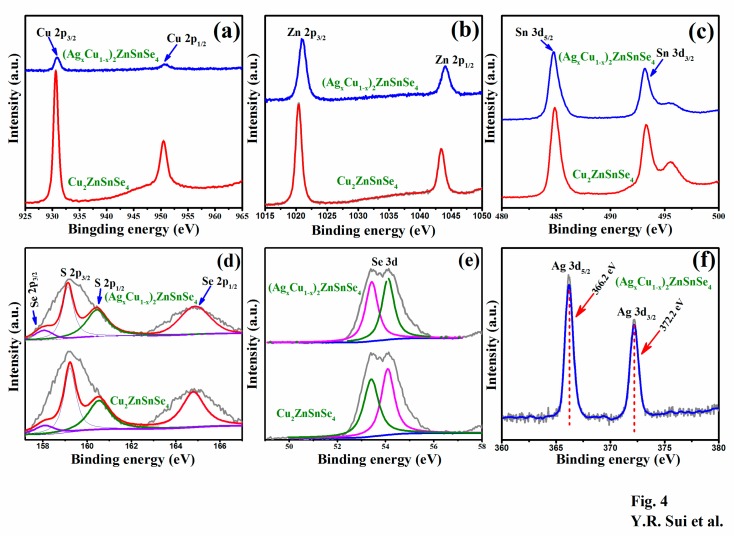
X-ray photoelectron spectroscopy (XPS) spectra of (**a**) Cu 2p, (**b**) Zn 2p, (**c**) Sn 3d, (**d**) S 2p, (**e**) Se 3d, and (**f**) Ag 3d for the CZTSSe and CAZTSSe (x = 0.1) alloy thin films.

For the sake of characterizing the valence states of component elements, the CAZTSSe (x = 0) and CAZTSSe (x = 0.4) films were performed by XPS measurement. Figure 4 displays the XPS profiles of the elements (Cu, Zn, Sn, S, Se, and Ag). Figure 4a shows the narrow scan XPS spectra of the Cu2p for CAZTSSe (x = 0) and CAZTSSe (x = 0.4) samples. Peaks at around 930.6 and 950.5 eV were ascribed to the Cu 2p_3/2_ and Cu 2p_1/2_, respectively. The splitting between 2p_3/2_ and 2p_1/2_ were 19.9 eV. This is in agreement with the standard splitting value of Cu^+^ [27]. Thus, Cu exists in the Cu^+^ state, which was obtained by reduction of Cu^2+^ during chemical reaction [27]. In addition, the intensity of the Cu 2p peak decreased with Ag doping, as displayed in Figure 4a, indicating that the relative content of the Cu in the CAZTSSe (x = 0.4) decreased compared with the undoped CZTSSe. This supported the conclusion about the replacement of Ag in Cu sites obtained from the XRD and Raman results. Two strong peaks located at around 1020.7 eV and 1043.8 eV appeared in Figure 4b. The binding energies were consistent with Zn 2p_3/2_ and Zn 2p_1/2_, respectively. The split orbit is 23.1 eV which was in agreement with the standard interval value of 22.97 eV, indicating Zn exists in Zn^2+^ oxidation state [28]. The XPS spectra of Sn 3d had two peaks at 494.72 eV and 486.22 eV with a split value of 8.5 eV, which was in agreement with the standard splitting value of Sn^4+^. It well supported the fact of +4 oxidation state for Sn in CAZTSSe (x = 0) and CAZTSSe (x = 0.4) films. This indicated that the Cu^2+^ was reduced to Cu^+^ and Sn^2+^ was oxidized to Sn^4+^ during the fabrication. In addition, a shoulder peak was found near the Sn 3d peak, which can be regarded as the Zn-Auger peak L3M45M45 [29]. The narrow scan XPS spectra of S are shown in Figure 4d. Based on the Gaussian–Lorentzian fitting, the binding energy values were estimated as 159.0, 160.1, 161.4, and 165.8 eV, which could be assigned to Se 2p_3/2_, S 2p_3/2_, S 2p_1/2_, and Se 2p_1/2_, respectively. The peaks at 160.1 and 161.4 eV were attributed to S 2p_3/2_ and S 2p_1/2_. The 2p peak of S was located in the range of 160–164 eV, indicating that it was in a sulfide state [30]. It could be seen from the Figure 4e that XPS spectra of Se 3d was fitted into two peaks, located at 53.4 eV and 54.1 eV, which were attributed to Se 3d_3/2_ and Se 3d_1/2_, respectively. The binding energy of the Se 3d was in agreement with previously reported binding energy of Se^2−^ for CZTSSe thin film [31]. The characteristic peaks with the binding energy of 834.2 eV and 851.0 eV displayed in Figure 4f were attributed to Ag 3d_5/2_ and Ag 3d_3/2_, respectively. The peak splitting of 16.8 eV indicated that Ag existed in the chemical state of Ag^+^ [21]. It gives a reflection that Ag was incorporated into CZTSSe and replaced the sites of Cu atom to form the CAZTSSe film. 

The chemical composition of CAZTSSe film (0 ≤ x ≤ 1) was characterized by EDS and shown in Table 1. According to the characterization results, the atomic percentages of Ag increased from 0 to 21.16, while for Cu it distinctly decreased from 21.01 to 0 with the x increasing. The results showed that as the Ag content was increased, the atomic percentages of Cu in CAZTSSe films decreased, which well supported the conclusion that Cu atoms were replaced by Ag atoms obtained from the XRD results. The atomic ratios of Ag/(Cu+Ag) and (Ag+Cu)/(Zn+Sn) are also listed in Table 1. It was found that atomic ratio Ag/(Cu+Ag) in CAZTSSe films was well adjusted by controlling the ratio of Ag/(Cu+Ag) in the precursor solution. The stoichiometric ratio of (Ag+Cu)/(Zn+Sn) showed that the CAZTSSe films had zinc-rich and copper-poor conditions. It has been reported that poor copper and rich zinc films can improve the cell performance of the CZTSSe device [32]. The Sn and anion contents fluctuated very little, which was mainly due to the lower annealing temperature (480 °C) during the annealing. The high efficiency CZTSSe devices generally need a higher annealing temperature (about 540 °C). This could result in the decrease in Sn content by the volatilization of SnSe_2_ and increase in relatively Cu and Zn content. And this will cause the increase of impurity phase and defects. It is worth mentioning that all the smooth and compact CAZTSSe films were obtained at the lower annealing temperature of 480 °C in the present work. Therefore, it can be concluded that introduction of Ag made the annealing temperature decrease, which avoided the volatilization of Sn and decreased the content of impurity phase and defects.

**Figure 5:**
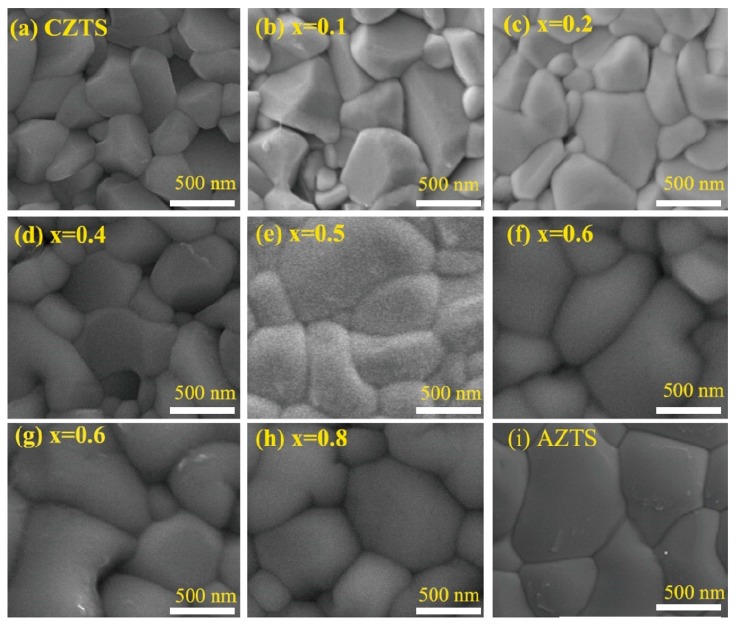
Scanning electron microscopy (SEM) images (**a**–**i**) of the CAZTSSe (0 ≤ x ≤ 1) alloy thin films.

The surface morphology of CAZTSSe films (0 ≤ x ≤ 1) is displayed in Figure 5. Here we characterized notable changes in the grain size upon Ag-incorporation. The undoped CZTSSe thin film surface consisted of grains with the order of nanometers, and had some cracks. With increasing x from 0.1 to 0.8, it was found that the CAZTSSe films displayed the smoother and more compact surface morphology. The obvious holes and cracks decreased with the increase of the Ag doping content, some larger grains with a size of 1–2 um appeared. It implied that the grain size of CAZTSSe film was improved, which was consistent with XRD result. In the literature previously reported, alkali metal could distinctly accelerate the grain size of CZTSSe and CIGS by forming quasi-liquid K_x_Se and Na_x_Se [33,34]. In our work, the increase of grain sizes could be attributed to the formation of liquid phase of Ag–Se binary compounds during selenization, and the Ag–Se binary compounds were considered as a selenium source which could promote grain growth [35]. The liquid Ag–Se binary compounds could promote the diffusion of constituent atoms and merge the CZTSSe grain boundary. The SEM image of AZTSSe thin films is shown in Figure 5i. It was clear that the AZTSSe thin films displayed the smooth and compact surface morphology, no obvious holes or cracks could be found, and the average grain size was up to 3 um.

The influence of Ag content on the optical band gap of CAZTSSe (0 ≤ x ≤ 0.4) samples was studied. And the optical measurement of CAZTSSe (0 ≤ x ≤ 0.4) films was characterized by UV–VIS–NIR. The band gap of CAZTSSe (0 ≤ x ≤ 0.4) films was obtained according to the absorption spectrum by Tauc’s equation:

*hυ* = *B*[(*hυ* − *E_g_*)^*n*^/*hυ*] (1)

where is the absorption coefficient, *B* is a constant, hυ is the photon energy. The value of “*n*” depends on the different types of semiconductor, taking the values of 1/2, 3/2, 2, and 3 when the transitions are direct allowed, direct forbidden, indirect allowed, and indirect forbidden, respectively. The film is regarded as more suitable for direct band gap energy material and hence *n* = 1/2 is employed for this work, the relationship between and band gap can be described as:

*hυ* = *B* (*hυ* − *E_g_*)^1/2^ (2)

**Figure 6:**
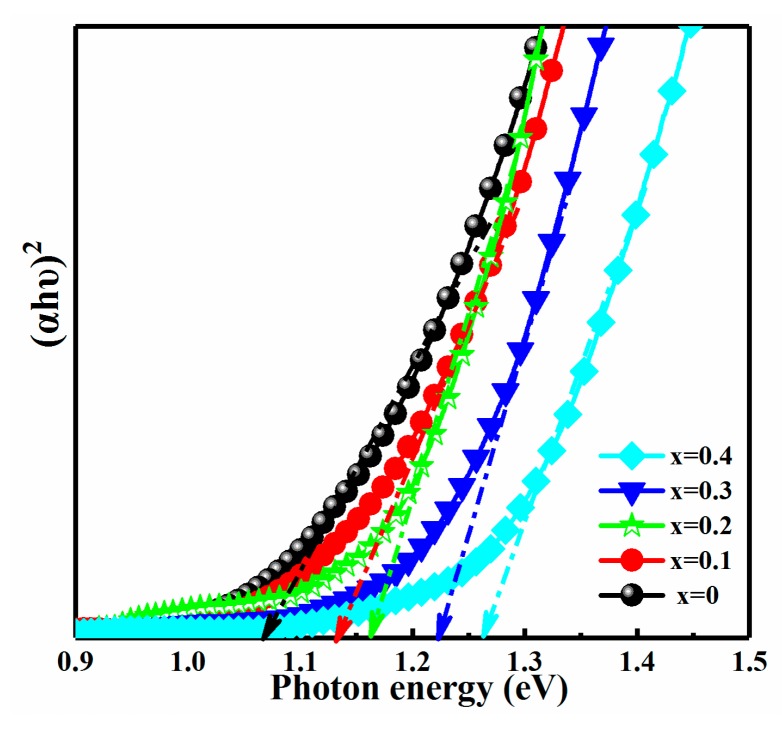
Plot of (*αhυ*)^2^ against hυ for the CAZTSSe (0 ≤ x ≤ 0.4) alloy thin films.

The *E_g_* can be acquired by extrapolating the linear portion of the curves (*αhυ*)^2^ as a function of hυ to intercept the energy x-axis. When (*αhυ*)^2^ = 0, *E_g_* = *hυ*. Figure 6 shows the (*hυ*)^2^ of the CAZTSSe (0 ≤ x ≤ 0.4) as a function of hυ, respectively. According the data in Figure 6 and Equation (2), the band gaps of CAZTSSe (x = 0, 0.1, 0.2, 0.3, 0.4) were calculated to be 1.07, 1.13, 1.16, 1.22, and 1.26, respectively. It could be found that the *E_g_* increased from 1.07 to 1.26 eV as x increased from 0 to 0.4. The increase of the band gap was well matched to previous reports [36]. The band gap of CAZTSSe film was decided according to the conduction band minimum (CBM) and valence band maximum (VBM) [37]. The CBM was determined by anti-bonding components of s-s and s-p hybridization between Sn^4+^ and Se^2−^, and the VBM was determined by the anti-bonding components of p-d hybridization between Se^2−^ (S^2−^) and Cu^+^ (Ag^+^) [13]. In this paper, the change of the band gap with Ag doping content may be attributed to the change of the VBM caused by the anti-bonding components of p-d hybridization between Se^2−^ and Cu^+^ (Ag^+^) [38].

**Figure 7:**
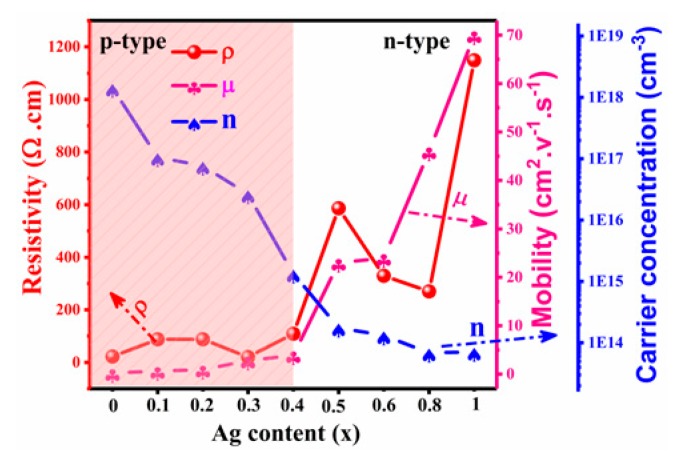
The electrical properties including the resistivity (ρ), carrier concentration (n), and mobility (μ) of the CAZTSSe (0 ≤ x ≤ 1) alloy thin films.

Figure 7 shows the electrical characteristics of CAZTSSe (0 ≤ x ≤ 1) films with different Ag contents. It can be seen that CAZTSSe (x = 0) film exhibited p type conductivity. Several literatures had indicated that Cu_zn_ defects are primarily the acceptor in CZTSSe thin films, which made CZTSSe thin films to show p-type conductivity [37,38]. When the x varied from 0.1 to 0.4, all samples showed p-type conductivity, and the carrier concentration uniformly reduced with increase of Ag-doped contents. Theoretical results indicated that deeper acceptor antisite Ag_Zn_ had higher formation energy and it will inhibit the formation of Cu_Zn_ antisite acceptor as the Ag doping content was increased in CAZTSSe film [13]. Therefore, the carrier concentration of the CAZTSSe films decreased as Ag doping content increased. The mobility increased as Ag doping content increased, indicating the reduced scatters in CAZTSSe film. The CAZTSSe at x = 0.4 displayed preferable p-type conductivity with a carrier concentration of 1.34 × 10^15^ cm^–3^ and a hall mobility of 3.9 cm^2^V^−1^s^−1^. As the x increased to 0.5, the conduction type of the CAZTSSe film transformed to n type. The results are in agreement with the theoretical forecast [13]. It had been reported that the conduction type of the AZTSSe film was n type, because antisite defects of the Zn instead of Ag site (Zn_Ag_) was primarily donor defect and had a relatively shallow level than Ag_Zn_ acceptor defect for Ag_2_ZnSnS_4_ film [13]. Therefore, as the Ag doping content increased, the conductive type of CAZTSSe conversed the weakly n-type.

**Figure 8:**
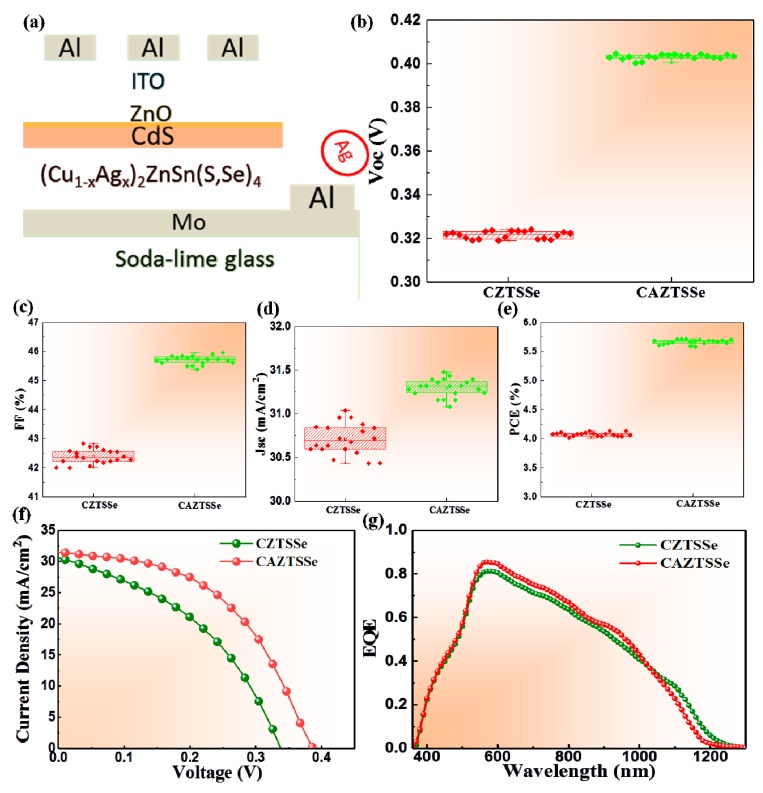
(**a**) The device schematic structure of CAZTSSe. (**b**–**e**) Device parameters statistics of the CZTSSe and CAZTSSe (x = 0.1) devices. (**f**) Current-voltage characteristics of the CZTSSe and CAZTSSe (x = 0.1) devices. (**g**) External quantum efficiency (EQE) spectra of the CZTSSe and CAZTSSe (x = 0.1) devices.

To further study the effect of Ag incorporation on device performance, CAZTSSe device the with structure of SLG/Mo/CAZTSSe/CdS/ZnO/ITO/Al was synthesized and is shown in Figure 8a. The average device performance parameters including V_oc_, short circuit current (J_sc_), fill factor (FF), and PCE of CZTSSe and CAZTSSe (x = 0.1) based on 18 devices are shown in Figure 8b–e. The results are average of 18 devices that provided a statistical consistency. According to the statistical data, a relationship can be observed between the device performance of CZTSSe and CAZTSSe (x = 0.1). J_sc_, FF, and V_oc_ has been improved, especially, V_oc_ witnessed an appreciable rising from 330 to 490 mV. Then, the average PCE was improved from 4.24 to 5.66%. The best device performance of CZTSSe and CAZTSSe are shown in Figure 8f and device parameters are also listed in Table 2. It was clear that J_sc_, V_oc_, and FF for CAZTSSe device was much higher than that of CZTSSe device and obtained a PCE of 5.95%. The series resistance (R_s_) was 1.2 Ω cm^2^ and shunt resistance (R_sh_) was 675.7 Ω cm^2^ for CAZTSSe cells, whereas R_s_ was 2.5 Ω cm^2^ and R_sh_ was 456.5 Ω cm^2^ for CZTSSe cells. The decrease in R_s_ and increase in R_sh_ is mainly due to the improved grain size and the decrease of Cu_zn_ concentration for CAZTSSe device. The decreased R_s_ and increased R_sh_ led to the increase of J_sc_ and FF. It could be found that a remarkable increase in V_oc_ of approximately 160 mV was obtained, which was significantly higher than previously reported result [21]. The improvement in device performance was attributed to the decreased Cu_Zn_ defects which could reduce the band tailing and led to large bending at the interface of CAZTSSe and CdS. To characterize photogenerated carrier collection, the external quantum efficiency (EQE) was measured and is presented in Figure 8g for the CZTSSe and CAZTSSe solar cells. EQE spectra showed a significant increase in the whole visible region for CAZTSSe device, which implied that recombination rate of photo-generating carriers for the CAZTSSe film is lower [39]. This improvement of EQE is in agreement with the increase in J_sc_ of CAZTSSe cells.

## 4. Conclusions

In summary, Ag has been introduced into Cu_2_ZnSn(S,Se)_4_ (CZTSSe) thin film to replace Cu partially and form (Cu_1−x_Ag_x_)_2_ZnSn(S,Se)_4_ (0 ≤ x ≤1) (CAZTSSe) alloy films by the combination of solution method and a rapid annealing technique. The effect of Ag doping on the CZTSSe film and device performance had been studied in detail. It was concluded that the replacement of some Cu sites by Ag occurs in CAZTSSe alloy films according to the result of structural characterization. The band gap of kesterite CAZTSSe is incessantly increased by adjusting the Ag-doped content, indicating that the CAZTSSe alloy films is a potentially applicable bandgap grading absorption layers material to obtain higher CZTSSe device. As the Ag doping content increase, the hole density was gradual decreased and the conduction type of the CAZTSSe film transformed to n type when the x increased to 0.5. Finally, we also found an increment in open circuit voltage (V_oc_) by 160 mV and an accompanying rise in device efficiency from 4.24 to 5.95%. The improvement was correlated to the improved grain size and decreased Cu_Zn_ antisite defects. In addition, the CAZTSSe cell displayed better charge collection performance based on the higher fill factor and PCE. Therefore, the doping of Ag is a potentially appropriate method to reduce the Cu_zn_ antisite defects of CZTSSe and improve efficiency of CZTSSe device.

## Figures and Tables

**Table 1:** Summary of the energy-dispersive X-ray spectroscopy (EDS) results of CAZTSSe (0 ≤ x ≤ 1) thin films with various Ag contents.

Sample	Cu (at%)	Zn (at%)	Sn (at%)	Ag (at%)	Se (at%)	S (at%)	(Ag+Cu)/Zn+Sn	Ag/(Cu+Ag)
**x = 0.0**	21.01	12.51	12.21	0.00	50.57	5.52	0.85	0.00
**x = 0.1**	18.91	12.81	12.12	2.02	50.23	4.93	0.84	0.09
**x = 0.2**	16.44	12.16	12.52	4.22	49.60	5.80	0.84	0.20
**x = 0.3**	14.73	12.25	12.52	6.25	50.56	4.64	084	0.29
**x = 0.4**	13.54	13.52	10.59	8.09	54.47	4.70	0.89	0.37
**x = 0.5**	12.42	12.03	12.38	9.30	50.77	5.22	0.88	0.42
**x = 0.6**	9.24	12.08	12.18	11.76	50.27	5.62	0.87	0.56
**x = 0.8**	5.46	12.09	12.46	15.54	50.29	5.76	0.85	0.74
**x = 1**	0.00	12.13	12.47	20.16	50.07	5.93	0.86	1.00

**Table 2:** Parameters of the device performance.

Device	Active Area	Voc (V)	Jsc (mA/cm^2^)	FF (%)	PCE (%)	Rs (Ω cm^2^)	Rsh (Ω cm^2^)
**CZTSSe**	0.19 cm^2^	0.33	30.28	43	4.24	2.5	456.5
**CAZTSSe (x = 0.1)**	0.19 cm^2^	0.49	31.41	46	5.95	1.2	675.7
